# To the Editors of the Medical and Physical Journal

**Published:** 1805-04-01

**Authors:** T. Girdlestone

**Affiliations:** Yarmouth


### To the Editors of the Medical and Physical Journal.

GENTLEMEN,

DR. HEBERDEN, in his elegant Epitome morborum puerilium, asks the following question: "Cum vero cernimus lac uberibus matris a Deo esse præparatum, cur non ars nostra primos faciat cibos lacti quam simillimos?" By this question, the Doctor probably intended an imitation of the degree of heat as well as of the nature of the food. But as no caution is given in his chapter De victu puerorum against heating food too hot, I am induced to submit a few words on this subject to the consideration of your readers. As the secretions of the human body are none of them hotter than the human blood, it is evident that nothing should go hotter than 98 degrees, of Fahrenheit's thermometer, into the stomach of an infant. But as this heat would be considered as cold by most nurses, who are accustomed to drink scalding hot tea, and who are foolish enough to think that there is no nourishment in any food which is not heated to nearly that degree, can it be wondered at, that so many infants have, within the first month, their mouths, throats, and stomachs, scalded into a thrush?

For these last eight or nine years, I have believed that the greatest numbers of aphtha which take place during the first month of infants are ulcerations from scalding hot food. The directions which I have given to mothers are, never to allow their nurse to feed the child without their first examining the warmth of the food, especially during the night, when the nurse, between sleeping and waking, is usually apt to overheat the food.

By attention to this precaution, my own children, and many others in this town, have escaped the thrush. That food impregnated with copper by standing too long in a silver vessel, or by containing any other saline or vegetable acrid, may also occasion aphtha, is not my intention to deny. And I am aware that Dr. Underwood has enumerated the too great heat of the food among the causes which may excite aphtha, though he has not limited the degree of heat at which an infant's food ought to be warmed.

I am, &c.

*Yarmouth, Feb.* 13, 1805. T. GIRDLESTONE.

